# Supplementary material for: Self-Reported Gum Bleeding, Perception, Knowledge, and Behavior in Working-Age Hong Kong Chinese—A Cross-Sectional Study
Source: Int J Environ Res Public Health. 2022 May 9;19(9):5749. doi: 10.3390/ijerph19095749 (PMC9103544; doi:10.3390/ijerph19095749)
Supplement: Supplementary file 1 [file ijerph-19-05749-s001.zip › ijerph-1658083-supplementary.pdf]

International Journal of
Environmental Research and Public Health

MDPI

Supplementary Information to Article

Self-Reported Gum Bleeding, Perception, Knowledge, and Behavior in Working-age Hong Kong Chinese—A Cross Sectional Study

Tsz Yung Wong, Yiu Cheung Tsang, Kim Wai Shadow Yeung and Wai Keung Leung *

Faculty of Dentistry, The University of Hong Kong, Hong Kong SAR, China; claratywong@connect.hku.hk (T.Y.W.); elvist@hku.hk (Y.C.T); skwyeung@hku.hk (K.W.S.Y.)

* Correspondence: ewkleung@hku.hk; Tel.: (+852-2859-0417)

2. Materials and Methods

2.3.1. Translated Survey Questionnaire (Original Version in Cantonese) [56]

I. *Interviewer self-introduction*

Good Morning/afternoon/evening, Mr./Ms., I am Ms./Mr. X. I am an interviewer from Public Opinion Programme of The University of Hong Kong. We are currently conducting a research on oral health conditions of Hong Kong people. We would like to invite you to participate in a telephone questionnaire. Your telephone number was selected from computer randomization, and all information collected in this survey is strictly confidential. Should you have any enquiries on this survey, please feel free to contact our supervisor Mr. RL/TY at our hotline 39XX-XX03. If you have questions about the rights as a research participant, please kindly contract the Human Research Ethics Committee for Non-Clinical Faculties, HKU at XX41-52XX. To ensure the validity of the data, our interview will be recorded and kept for 6 months for internal reference only. Shall we start the interview now?

☐ Yes
☐ No → the interview is completed. Thank you, goodbye.

[S1] Is your home phone number XXXX-XXXX?

☐ Yes
☐ No (skip to end)

II. *Identification of the interviewee*

[S2] Our survey subject has to be a Hong Kong resident who aged 25-60 years, had experience on bleeding gums (including bleeding during brushing, flossing, using interdental brushes, during eating or using toothpick, but excludes bleeding due to tooth exfoliation, accident or damage to teeth or gum). May I know how many family members in your household meet our targeted group? [If none of the household member is qualified to be interviewee, end of interview. Thank you, goodbye.]

☐ Yes, there is one → start interviewing [If the one on the phone is not qualified to be our subject, please invite another qualified family member to the phone and repeat the self-introduction]
☐ More than one, ___ people 【enter the actual number】→ [S3]
☐ No → end of interview Thank you, goodbye.
☐ Interviewee refuses to answer → end of interview. Thank you, goodbye.

[S3] Since there are more than one family members who can be a survey participant, we hope that all of them have equal chances to be interviewed. May I invite the one whose next birthday is the closest to be interviewed? (Interviewer can make an example "Is there any one whose birthday is in April or will be having their birthday within coming three months?")
[To ensure the validity of the data, our interview will be recorded for internal reference only.]

Shall we start the interview?

☐ Yes, the one who pick up the call is the selected interviewee → start interviewing
☐ Yes – the selected interviewee is another family member [interviewer repeat the self-introduction] → start interviewing
☐ The selected interviewee is not at home/unavailable [please ask for an available time for interview]
☐ No, family member refuses to answer → end of interview. Thank you, goodbye.
☐ No, the selected interviewee refuses to answer → end of interview. Thank you, goodbye.

III. Questionnaire after successful participant engagement

A. Oral Hygiene practice

1. Have you been using the following method to clean your teeth daily? (readout a computer-generated random sequence of the following three items, multiple responses allowed)

- ☐ Tooth brushing with dentifrice
- ☐ Flossing/interdental brushing
- ☐ Using mouth-rinse
- ☐ None of the above (Go to Q3)
- ☐ Don't know
- ☐ Refuse to answer

2. The duration of tooth brushing when wake up in the morning or before going to bed (exclude flossing and/or interdental brushing) (*no answer provided, only one response allowed*)

	**After wake up in the morning**	**Before going to bed at night**
Never brush		
< 10 seconds		
10 - 29 seconds		
30 - 59 seconds		
1 - 1 minute 59 seconds		
2 - 2 minutes 59 seconds		
≥ 3 minutes		
Don't know		
Refuse to answer		

B. Gum bleeding experience

The following are questions regarding gum bleeding, for instance, bleeding when brushing, interdental brushing or flossing; or when bleeding is detected while you eat or using toothpicks. Please ignore bleeding when your head/mouth/tooth is traumatized, including the same day after tooth extraction.

3. Please indicate the age when you first experience gum bleeding? (*no answer provided, only one response allowed*)

- ☐ ≤ 10 years
- ☐ 11 - 15 years
- ☐ 16 - 20 years
- ☐ 21 - 25 years
- ☐ 26 - 30 years
- ☐ 31 - 35 years
- ☐ 36 - 40 years
- ☐ 41 - 45 years
- ☐ 46 - 50 years
- ☐ ≥ 51 years
- ☐ Don't know / can't remember
- ☐ Refuse to answer

4. Over the past 12 months, did you experience gum bleeding? If so, on average how often it comes? (*no answer provided, only one response allowed*)

- ☐ Yes, ≥ 2 times a week
- ☐ Yes, 1 time a week
- ☐ Yes, 2 - 3 times a month
- ☐ Yes, 1 time a month
- ☐ Yes, 1 time 2 - 3 months
- ☐ Yes, 1 time every ≥ 4 months
- ☐ Yes, refuse to give details/ don't know / can't remember
- ☐ No (Go to Q6)
- ☐ Don't know
- ☐ Refuse to answer

5. Over the past 12 months, did you experience gum bleeding over a consecutive 3 days? If so, on average how often it comes? (*no answer provided, only one response allowed*)

- ☐ Yes, 2 times a week
- ☐ Yes, 1 time a week
- ☐ Yes, 2 - 3 times a month
- ☐ Yes, 1 time a month
- ☐ Yes, 1 time 2 - 3 months
- ☐ Yes, 1 time every ≥ 4 months
- ☐ Yes, refuse to give details/ don't know / can't remember
- ☐ No (Go to Q6)
- ☐ Don't know
- ☐ Refuse to answer

6. As far as you can recall, what was the longest duration of consecutive (> 1 day) gum bleeding that ever happened to you? (*no answer provided,* multiple responses allowed)

- ☐ 2 days
- ☐ 3 - 6 days
- ☐ 1 week
- ☐ 2 - 3 weeks
- ☐ 4 weeks
- ☐ 1 - 2 months (5 - 8 weeks)
- ☐ 3 - 6 months
- ☐ 7 - 12 months
- ☐ ≥ 1 year
- ☐ Never has consecutive gum bleeding
- ☐ Don't know/can't remember
- ☐ Refuse to answer

7. According to your understanding, which of the followings statements is related to gum bleeding? (Interviewer to read out a random computer-generated sequence of the followings; *only one response allowed per statement*)

	**Yes**	**No**	**Don't know**	**Refuse to answer**
Consumption of *yeet-hay** food (e.g. had hot-pot)				
In bad mood/under pressure				
Lack of sleep/work or play overnight				
smoking				
Poor oral hygiene or with gingivitis/periodontitis				
pregnant				

*Heaty

8. Have you ever come across gum bleeding in association with the following situations? (Interviewer to read out a random computer-generated sequence of the followings; *only one response allowed per statement*).

	**Yes**	**No**	**Don't know**	**Refuse to answer**
Consumption of *yeet-hay** food (e.g. had hot-pot)				
Poor health condition (e.g. flu, sore throat, on medications)				
In bad mood/under pressure				
Lack of sleep/work overnight				
Poor oral hygiene/not brushing well				

*Heaty

9. Have you ever tried the following ways to manage your bleeding gum? (Interviewer to read out a random computer-generated sequence of the followings; *only one response allowed per statement*)

	Yes - often	Yes - occasional	Never	Don't know	Refuse to answer
Attend dentist					
Attempt to brush better/flossing/interdental brushing					
Ignore, await condition automatically gets better					
Drink *heat* purging herbal Chinese medicine/stop *yeet-hay** food intake					
Rinse mouth with saline					
Avoid brushing the bleeding area					

*Heaty

10. Based upon your understanding, what are the consequences if gum bleeding was ignored? (no answer provided, multiple responses allowed)

- ☐ Gum inflammation
- ☐ Periodontitis
- ☐ Gum boil/abscess
- ☐ Tooth loss
- ☐ Bad breath
- ☐ Other, please specify: ________
- ☐ No consequences
- ☐ Don't know / can't remember
- ☐ Refuse to answer

11. Do you currently suffer from the following dental problem? (Interviewer to read out a random computer-generated sequence of the followings; *only one response allowed per statement*)

	Yes	No	Don't know	Refuse to answer
Periodontitis				
Mobile/drifted tooth				
Receding gum/tooth become longer				
Gum swelling/pain				
Sensitive tooth				
Extraction/exfoliation of permanent tooth				

C. Dental experience

12. Are you a regular dental attender? If so, how often? (*no answer provided, only one response allowed*)

- ☐ Yes, once every 6 months
- ☐ Yes, once every year
- ☐ Yes, once every 2 years
- ☐ Yes, once > 2 years
- ☐ Only when there is a problem
- ☐ Never attended any dentist (Go to Section D)
- ☐ Don't know / hard to say
- ☐ Refuse to answer

13. Indicate for each of the following statement given, in what ways it correctly describes your reaction? (Interviewer to read out a random computer-generated sequence of the followings; *only one response allowed per statement*)

	Totally agree	Disagree	Don't know/ hard to say	Refuse to answer
I become nervous when the dentist invites me to sit down in the chair				

I want to walk out of the waiting room the moment I think the dentist will not explain what s/he is going to do in my mouth				
As soon as the dentist gets his/her needle ready for the anesthetics, I shut my eyes tight				
In the waiting room, I sweat or freeze when I think of sitting down in the dentist's chair				
When I am sitting in the dentist's chair not knowing what is going on in my mouth, I break in a cold sweat.				

D. *Work, finance, life/family pressure*

14. On a 0 - 10 point scale, how would you rate your current work, finance, life/family pressure? (0 = no pressure, 10 = maximum pressure, 5 = 50/50)

(a) work pressure: ___ [exact quote from interviewee]
☐ Don't know/hard to say
☐ Refuse to answer

(b) Financial pressure: ___ [exact quote from interviewee]
☐ Don't know/hard to say
☐ Refuse to answer

(c) life/family pressure: ___ [exact quote from interviewee]
☐ Don't know/hard to say
☐ Refuse to answer

E. *Personal particulars*

We want to gather some of your background character so as to analysis how our different background may influence your opinions. The following information collected will be used exclusively for this survey and would be kept secret. In case you like to refuse answering any part of the questions, please just let us know.

15. Gender（interviewer to record）
☐ M
☐ F

16. Age
(a)
☐ ______ [exact quote from interviewee]
☐ Refuse to answer

(b)【only for those refused to give exact age】
Age range [interviewer can verbalize the ranges]
☐ 25 - 29 years
☐ 30 - 34 years
☐ 35 - 39 years
☐ 40 - 44 years
☐ 45 - 49 years
☐ 50 - 54 years
☐ 55 - 60 years
☐ Refuse to answer

17. Do you smoke?
☐ Yes
☐ Yes I did, quitted
☐ Never
☐ Refuse to answer

18. Education level

☐ Primary or below
☐ Form 1 - 3 (junior)
☐ Form 4 - 5 (senior)
☐ Form 6 (HKDSE*/pre-university/gymnasium)
☐ Form 7 (matriculation)
☐ non-degree tertiary education
☐ Bachelor degree
☐ Master degree
☐ Doctoral degree or above
☐ Refuse to answer

*Hong Kong Diploma of Secondary Education

19. Work status

☐ Full-time
☐ Part-time
☐ Unemployed (skip to end)
☐ Unemployed – student (skip to end)
☐ Unemployed – retired (skip to end)
☐ Housewife (skip to end)
☐ Other, please specify: ________
☐ Refuse to answer

20. Monthly income [For respondent in full-/part-time employment only]

☐ ≤ HK$5,000
☐ HK$5,001 - 10,000
☐ HK$10,001 - 20,000
☐ HK$20,001 - 30,000
☐ HK$30,001 - 40,000
☐ HK$40,001 - 50,000
☐ ≥ HK$50,001
☐ Refuse to answer

This is the end of the survey, many thanks for your participation. In case of any queries regarding this interview, please contact our survey Supervisors at tel: 39XX-XX03, or if you have questions about the rights as a research participant, please contact the Human Research Ethics Committee, the University of Hong Kong at tel: XX41-52XX within office hours, Goodbye.

3. Results

3.1. Participants' Background

Table S1. Additional backgrounds, habit, gum bleeding experience, and perceptions of study participants (n = 516).

	Gum bleeding over last 12 months?				
	**No (n =195)**		**Yes (n = 321)**		***p*-value[1]**
**Smoking habit**					
- No	163	(83.6%)	277	(86.3%)	0.477
- Yes	32	(16.4%)	44	(13.7%)	
**Work status**					
- Full-time	119	(61.0%)	193	(60.1%)	0.760
- Part-time	17	(8.7%)	32	(10.0%)	
- Unemployed - student/retired	19	(9.8%)	24	(7.5%)	
- Housewife	40	(20.5%)	72	(22.4%)	
**Monthly income (HK$)[2]**	n = 123		n = 199		

- 10,000 or below	31	(25.2%)	50	(25.1%)	0.943
- 10,001 - 20,000	43	(35.0%)	67	(33.7%)	
- 20,001 - 30,000	25	(20.3%)	46	(23.1%)	
- 30,001 or above	24	(19.5%)	36	(18.1%)	
**Asymptomatic dental attendance**[3]	109	(56.2%)[4]	184	(57.3%)	0.873
**Dental anxiety (I agree with the following phrase)**[5]					
- I become nervous when the dentist invites me to sit down in the chair	95	(48.7%)	132	(41.1%)	0.336
- I want to walk out of the waiting room the moment I think the dentist will not explain what s/he is going to do in my mouth	37	(19.0%)	50	(15.6%)	0.380
- As soon as the dentist gets his/her needle ready for the anaesthetic, I shut my eyes tight	94	(48.2%)	173	(53.9%)	0.245
- In the waiting room, I sweat or freeze when I think of sitting down in the dentist's chair	21	(10.8%)	50	(15.6%)	0.160
- When I am sitting in the dentist's chair not knowing what is going on in my mouth, I break into a cold sweat	25	(12.8%)	56	(17.4%)	0.202
**First noticed gum bleeding at age (years, median)**	16 - 20	(21.0%)	11 - 15	(25.9%)	0.166
**Over past 12 months, the longest duration of daily gum bleeding**					
- 1 day	-		180	(56.1%)	
- 2 days	-		69	(21.5%)	
- 3 - 6 days	-		55	(17.1%)	
- ≥ 1 week	-		17	(5.3%)	
**Perceived outcomes if gum bleeding not properly attended to/managed:**					
- Gingivitis	51	(26.2%)	86	(26.8%)	0.955
- Periodontitis	93	(47.7%)	162	(50.5%)	0.603
- Tooth loss	37	(19.0%)	69	(21.5%)	0.565
- Don't know	14	(7.2%)	4	(1.2%)	-

[1] Fisher's Exact Test/Chi-square Test. [2] Only for participants who reported under full-/part-time employment; Hong Kong (HK)$ 7.8 = US$ 1.0; 59/96 respondents not experienced/experienced gum bleeding over past 12 months refuse to provide answer to the question. [3] Dental attendance recategorized into asymptomatic attendance (once every 6-months to >2 years) or symptomatic attendance (never/only when there is a problem) [26]. [4] $n = 194$, one participant reported 'don't know' was excluded. [5] Multiple answers allowed so total does not add up.

Age, gender and self-reported monthly income were compared to the then Hong Kong Census data (Tables S2-S4) [57,58]. No significant difference was detectable concerning the age categories or gender proportion of the CATI participants vs. that of the 2014 local census reports (Table S2, Chi-square tests, $p > 0.05$). The similar was observed concerning the reported distributions of participants with employment (Table S3) as well as their reported monthly income ranges (Table S4).

Table S2. Age and gender distributions of CATI participants with reference to local population.

Age group (years)	Participants (%)	Population[1] (%)	Ratio (%)
25 – 29	54 (10.7)	515,900 (11.3)	0.0105
M	21	224,200	

F	33	291,700	
30 - 39	97 (19.1)	1,141,100 (25.2)	0.0085
M	39	459,900	
F	58	681,200	
40 - 49	146 (28.8)	1,168,600 (25.8)	0.0125
M	52	497,900	
F	94	670,700	
50 - 60	210 (41.4)	1,710,600 (37.7)	0.0123
M	66	829,500	
F	144	881,100	

[1]Census and Statistics Department [57].

Table S3. Distributions of CATI participants' self-reported full-/part-time employment.

Age group (years)	Participants (%)	Population[1] (%)	Ratio (%)
25 - 34	81 (22.4)	775,500 (28.3)	0.0104
35 - 44	93 (25.8)	767,700 (28.0)	0.0121
45 - 54	139 (38.5)	746,900 (27.3)	0.0186
≥ 55	48 (13.3)	448,100 (16.4)	0.0107

[1]Census and Statistics Department [57].

Table S4. Distributions of CATI participants' self-reported monthly income (HK$).

Age group (years)	Present study (range)	Population (median)[1]
25 - 34	10,001 - 20,000	15,500
35 - 44	20,001 - 30,000	17,200
45 - 54	10,001 - 20,000	15,000
≥ 55	10,001 - 20,000	12,200

[1]Census and Statistics Department [58].

3.4. Factors Associated with Self-reported Gum Bleeding Over Past 12 Months

3.4.1. Data Recategorization

After data collation and cross checking, the following independent variables were recategorized because of small *n* observed in certain subcategories: i) longest duration of gum bleeding into 1 day, 2 days, 3-6 days or ≥ 1 week; ii) dental attendance was collapsed into either any attendance (asymptomatic attendees), or only when there is a problem/never (symptomatic attendees, [26]); iii) education levels into either secondary/below or tertiary/above; iv) smoking habit into either yes or never/quitted; v) unemployment status due to being student or retirement was

combined; vi) monthly income into HK$ ≤ 10,000, 10,001-20,000, 20,001-30,000, or ≥ 30,001; regarding vii) self-reported 'manage own gum bleeding by', the responses were collapsed into either yes (yes, often/occasional) or never/don't know.

Data Availability Statement: An summary highlighting the raw CATI data was presented at June 2014 Monthly meeting, The Hong Kong Dental Association [59].

References (continued from main text)

56. Chung, T.Y.R.; Pang, K.K.L.; Lee, W.W.Y.; Lee, K.W.; Tai, E.C.F.; Yu, C.-H. *Hong Kong Population Oral Health Opinion Survey*; Public Opinion Programme, the University of Hong Kong: Hong Kong, China, 2014. Available online: https://www.hkupop.hku.hk/english/report/gumBleeding2014/content/resources/qre.pdf (accessed on 10 March 2022). (In Chinese).
57. Census and Statistics Department. *Hong Kong Annual Digest of Statistics*; Census and Statistics Department: Hong Kong, China, 2015. Available online: https://www.statistics.gov.hk/pub/B10100032015AN15B0100.pdf (accessed on 10 March 2022).
58. Census and Statistics Department. *2014 Report on Annual Earnings and Hours Survey*; Census and Statistics Department: Hong Kong, China, 2015. Available online: https://www.statistics.gov.hk/pub/B10500142014AN14B0100.pdf (accessed on 10 March 2022).
59. Leung, W.K. Periodontal health: Who's responsibility? *Hong Kong Dent. Assoc. Newsletter* **2014**, *4*, 8–10. Available online: https://www.hkda.org/newsletter/2014/v4/2014v4_p8_10.pdf (accessed on 10 March 2022).
